# Supplementary figures and images for: Intracellular presence of Helicobacter pylori antigen and genes within gastric and vaginal Candida
Source: PLoS One. 2024 Feb 8;19(2):e0298442. doi: 10.1371/journal.pone.0298442 (PMC10852334; doi:10.1371/journal.pone.0298442)

Fig2a

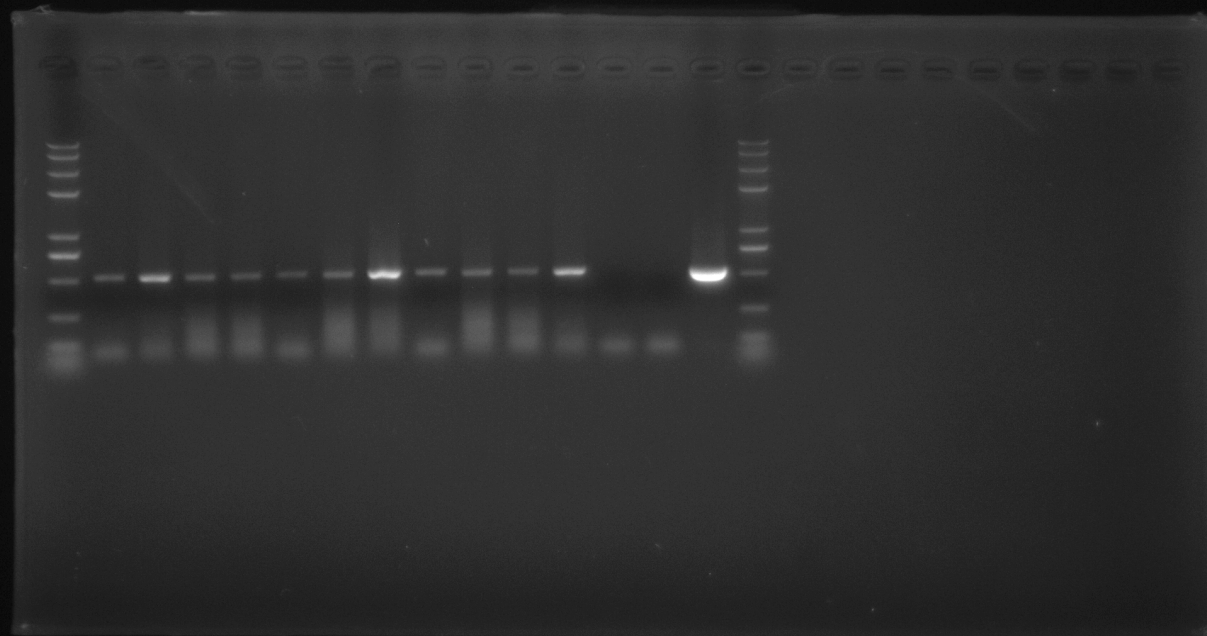

Fig2b

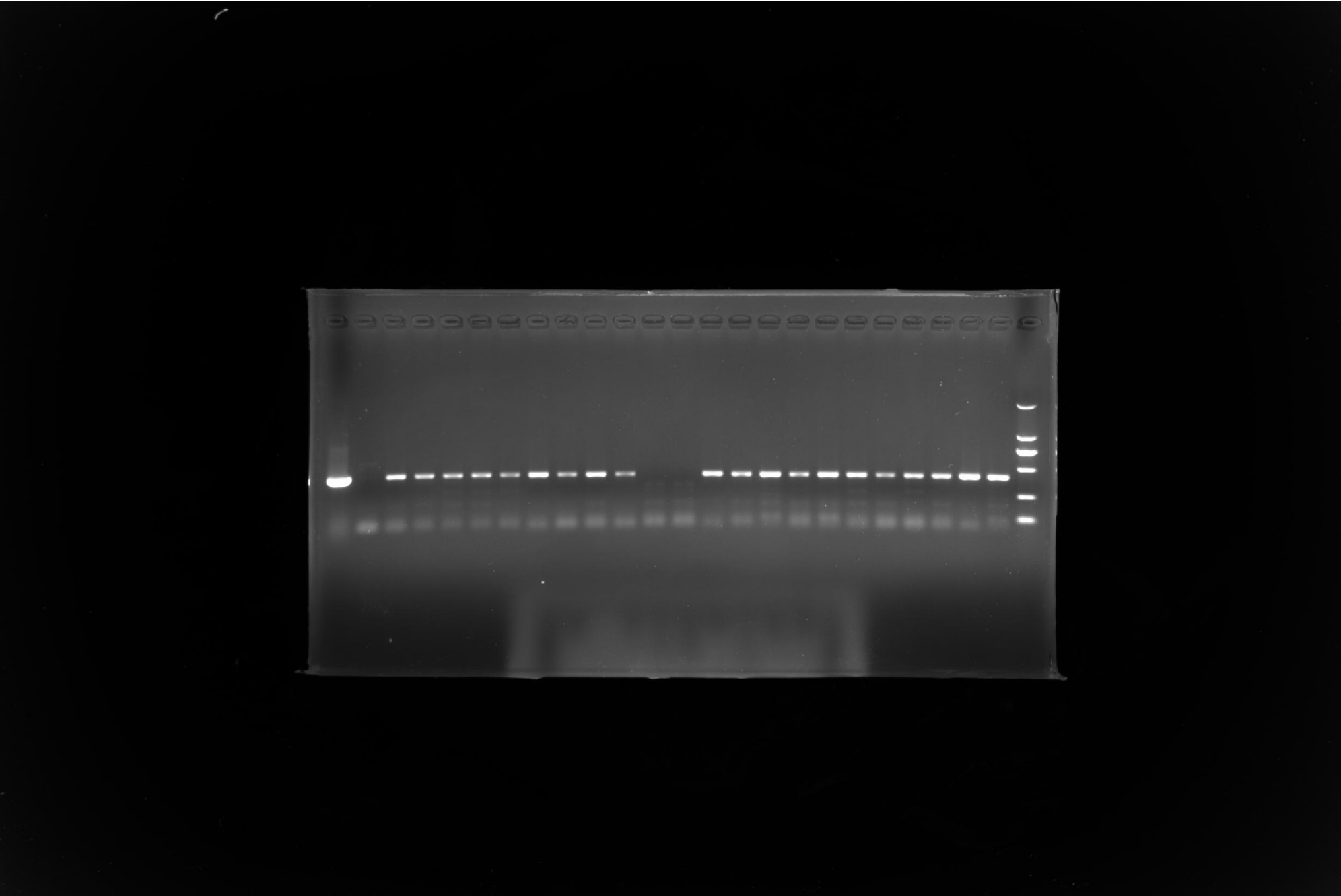

Fig3

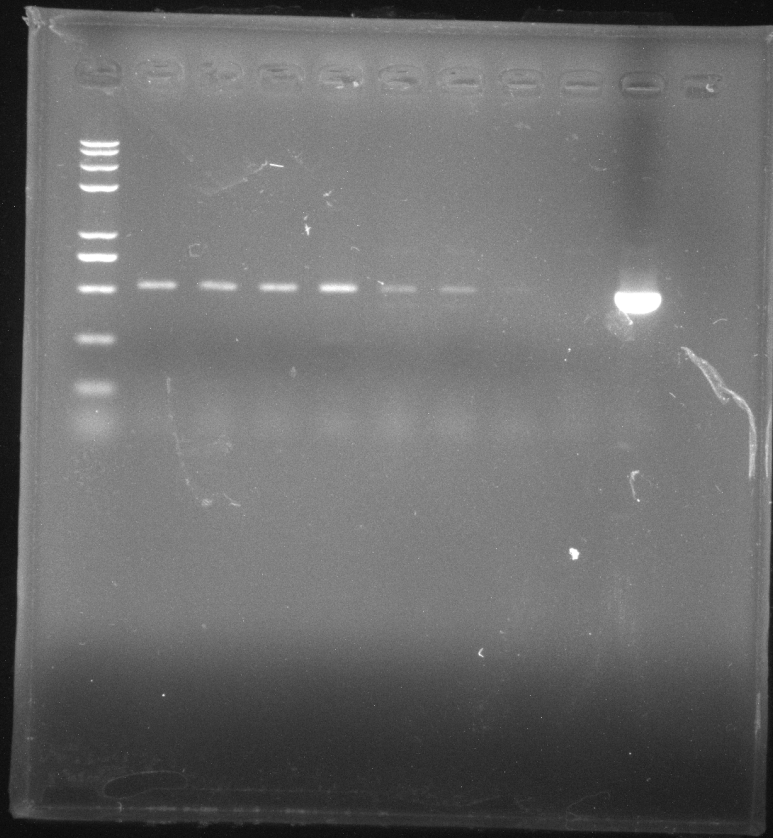

Supplement: S1 Fig — (PDF) [file pone.0298442.s001.pdf]
