# Supplementary material for: Intracellular presence of Helicobacter pylori antigen and genes within gastric and vaginal Candida
Source: PLoS One. 2024 Feb 8;19(2):e0298442. doi: 10.1371/journal.pone.0298442 (PMC10852334; doi:10.1371/journal.pone.0298442)
Supplement: Attachment — Submitted filename: PLOS- review July 2023.docx [file pone.0298442.s004.docx]

**PONE-D-23-17764**

Reviewer’s Comments

Intracellular presence of H. pylori antigen and genes within gastric and vaginal Candida

**Abstract:**

This section needs to be organized in 4 parts; Background (including the aim of study), Methods, Results and Conclusions. Results should be organized according to methods and both sections need more details. Authors should differentiate between the “presence” and “internalization” of H. pylori mentioned in the aim and conclusions of the study, respectively.

**Introduction:**

Lines 63-68 contradict the presence of H. pylori in gastrointestinal tract, including the oral cavity. According to several reports mentioned in the present study, occurrence of H. pylori has been demonstrated in the oral cavity by detection of H. pylori-specific genes. Furthermore, in the last line of conclusions in the discussion section, authors concluded that H. pylori transmission occurs during vaginal delivery and through the oral cavity of newborn. That means that even Candida yeast (as a shelter for H. pylori) has to enter the gastrointestinal tract of mother or child through the portal of oral cavity. Accordingly, in the present study, detection of H. pylori genes in Candida isolates from oral cavity of mothers and newborns could help to better clarify the results. However, a similar but more elaborated study has been performed and the results have been published in 2013 (reference # 24).

Line 68-69 is confusing and needs to be revised and supported by a reference. Line 73 is confusing. Line 77-78 must be clarified. Lines 76-84 must be included in one paragraph as the aim of study.

Above all these, this study lacks the literature review of previous studies about the intracellular life of H. pylori inside the vacuole of Candida yeast (isolated from different sources including oral cavity, stomach, vagina and a variety of foods) that have been published since 1998. According to these previously published reports, the story began when Candida yeast was isolated along with H. pylori from the cultures of gastric biopsies. Accordingly, if authors do not wish to acknowledge the previous studies, they need to explain how they came to the idea of studying the presence of H. pylori inside the yeast? Although they have used several of them in discussion section but mostly with wrong interpretations.

**Methods:**

This section needs major revisions due to inconsistency in the number of patients and type of samples. It is not clear why the number of samples in different groups is not equal. Use of 2 gastric yeasts as the representative of yeasts of gastrointestinal tract is not enough for conclusive results. Some methods like Live/Dead bacterial staining is too long and some like UBT has not been described. Patients’ information like age and type of gastric diseases is missing. Results of Candida identification and their correlation with other results were not considered in results and discussion sections. The relationship between type of Candida species and detection of intracellular H. pylori genes or observing BLBs has not been considered. What was the origin of the reference C. albicans (ATCC 10231)? The transmission electron microscopy section needs more details. This sentence is not clear and needs explanation: “*Candida* was treated with 32 μg/mL amphotericin B at 37°C shaking at 120 r/min for 24 h [28] ”. What was the purpose of using amphoterici B? For detection of urease activity in Candida: Did you detect this activity in all the yeasts positive for H. pylori? There is no description of recruited yeasts. A reference is needed for SDA+40% urea. Why 40% urea was used? What was the pH of the medium?

**Results:**

Every patient that carried yeast with or without positive result of H. pylori 16S rDNA detection must be classified according to: The type of sample from pregnant or non-pregnant women, the identity of isolated Candida (by CHROMagar), observation of intracellular bacteria (by light microscopy) and live bacteria (by Live/Dead stain), results of *cagA* and *ureA* detection, UBT and serology. Otherwise, inconsistency in the number and type of samples in the present study is totally confusing and will not allow reaching any conclusive results. Accordingly, information presented as figure 1 and Table 2 are confusing rather than being informative.

These two sentences need to be clarified:

1- “Furthermore, among *H. pylori-*negative patients, only one vaginal *Candida* isolate lacked BLBs and was negative for *H. pylori* 16S rDNA and *ureA* (Fig 1). These results indicate that *H. pylori* 16S rDNA was present in Candida isolates with BLBs. Individuals who provided such specimens were therefore infected with *H.pylori”*.

To correlate the detection of 16S rDNA or intracellular bacteria inside the vaginal yeast to H. pylori infection in the stomach or positive IgG antibody, strong explanations and supporting references are needed. Results of this study do not support this conclusion.

2- “Intracellular presence of *H. pylori*-specific genes within *Candida* subcultures”.

This must become clear in this study that why the authors subcultured yeast isolates more than 10 times. These subsequent subculturings (on SDA+ chloramphenicol) must have been performed at the beginning of isolation of yeasts and from then on, there is no need to subculture more than 10 times because the primary purpose should have been removing any probable extracellular bacterial contamination. According to previously published reports, persistent intracellular existence of H. pylori in yeast is an evolutionary phenomenon that cannot be changed by subcultures. This important issue has been totally ignored in this study.

- Results of electron microscopy are missing.

- What was the difference between BLB-positive and BLB-negative Candida yeasts?

Figure 4: Light microscopy: The images are so small and unclear that one cannot differentiate between bacterial cell and other intracellular entities. Arrows do not really point to any clear entity. These photographs do not show any convincing results.

Figure 5: Live/Dead stain: There is no need to describe the mechanism of staining in the legend. Arrows in B could point to yeast’s nucleus or mitochondria which generally stain green. There is no mention of killing yeast in method section, also no comment on the control yeast that stained green.

Figure 6: Micrographs show that yeasts’ specimens were not properly prepared. They show no details of intracellular structures. “High electron density body” is confusing and needs to be explained.

Fig 7: Photograph C must be replaced by a better one with higher magnification.

Figures in this section that are the main part of this study fail to show convincing results .

**Discussion:**

There are several major misunderstanding and misuse of references, also a wrong conclusion as the followings:
Line 249-250: Need a reference.

Line 252-253: Authors need to specify transmission to newborns’ oral cavity through vaginal delivery .

Line 256: The importance of *cagA* positivity has not been discussed.

Line 261-262: “H. pylori- specific nucleic acids” is not consistent with other parts of the manuscript.

Line 272-273: Use of amphotericin must be explained.

Line 278-279: References Burgers et al. 2008, Siavoshi &Taghikhani 2013 and Salmanian et al. 2008 are not related and have been misused.

Line 284-287: Are these related to your study or need a reference. In this study only two gastric yeasts were used that is not convincing and do not correlate with vaginal yeasts.

Line 288-292: Not included in the method and results of this manuscript. Furthermore, references are not relevant and have been misused.

Line 294-299: These lines are not related to the study and have been used by mistake.

Line 300-304: Totally misinterpreted and the references are not related.

**Conclusion:**
- There is no interpretation to show the importance of detection of *cagA* or *ureA* genes in H. pylori-positive yeasts. Also, no description for the repeating sentence of “H. pylori was detectable in Candida isolates at the end of 10th subculture”.

The last sentence in conclusion: “Candida can act as a reservoir of H. pylori in the gastrointestinal tract of women, allowing the bacteria to migrate through the intestine to the vagina, eventually infecting newborns during delivery”, does not correspond to the aim of study and the title of the manuscript. Gastrointestinal tract includes oral cavity, stomach and intestine. Therefore, this conclusion is not correct because there are no samples from the oral cavity and feces of mothers and oral cavity of newborns.

**Reviewer’s overall comment:** **This manuscript is not acceptable.**
This manuscript looks like a thesis that needs substantial corrections. Introduction and aim of study need to be organized according to an extensive literature review on the basic science of bacteria (prokaryote)-yeast (eukaryote) interactions. It needs more consistent and reliable methods, specially sampling. Results are disintegrated and non-coherent, photographs are the main weakness of the results. Discussion includes many mistakes, wrong interpretations and misuse of references. This manuscript is a weak version of the previously reported studies and thus is devoid of any novelty.
