# Supplementary material for: Intracellular presence of Helicobacter pylori antigen and genes within gastric and vaginal Candida
Source: PLoS One. 2024 Feb 8;19(2):e0298442. doi: 10.1371/journal.pone.0298442 (PMC10852334; doi:10.1371/journal.pone.0298442)
Supplement: Attachment — Submitted filename: Response to Reviewers.docx [file pone.0298442.s005.docx]

**Response to reviewers**

**Title:** Intracellular presence of *Helicobacter pylori* antigen and genes within gastric and vaginal *Candida*

**ID:** PONE-D-23-17764

We wish to thank the editor and referee for their valuable and constructive comments, which have helped us to improve our manuscript. We have carefully addressed each of the comments and hope that our revised manuscript will meet with your approval. The revised text is shown in the manuscript. Our point-by-point responses to the comments are detailed below.

**Response to the** **reviewer’s comments:**

**Reviewer #1:**

*1. The power analysis should be done for the patient number. The number of patients that is mentioned as 50 is enough or not? And the picture resolution is not good enough to publish.*

**Response:** In this study, 42 female patients were included, in addition to 50 pregnant women.

One *Candida* strain was isolated from each vaginal discharge sample of a pregnant woman, yielding 50 isolates. It was statistically significant.

We are about to resubmit pictures that meet the journal's requirements.

**Reviewer #2:**

*1. In the results, the authors though have submitted H. pylori sequences to the GenBank with accession numbers, failed to add a phylogenetic tree that illustrates their findings. They also try to discuss this without much proof in the results. I suggest they provide proof if they have the results as a figure in their results. The authors should also clarify their conclusions by been exact about the helicobacter genes they detected using PCR.*

**Response:**

In this study, the specific genes of *H. pylori* (*16S rDNA, cagA, ureA*) were amplified from *Candida* by PCR, and the sequence was confirmed to be *H. pylori* by BLAST alignment. In addition, we had submitted *H. pylori* sequences to the GenBank. Some sequences of *H. pylori* 16S rDNA (accession no. ON545841, ON545842 and ON631242) and *cagA* had been submitted to GenBank (accession no. OM779116, OM779117 and OM812999).

**Reviewer #3:**

*1.* *Abstract:*

*This section needs to be organized in 4 parts; Background (including the aim of study), Methods, Results and Conclusions. Results should be organized according to methods and both sections need more details. Authors should differentiate between the “presence” and “internalization” of H. pylori mentioned in the aim and conclusions of the study, respectively.*

**Response:** The abstract is organized in four sections: background, methods, results, and conclusions. The results are organized according to method, with more details in both sections. The “presence” refers to the detection of *H. pylori* antigens and specific genes in *Candida* cells by various methods. The “presence” in the aim of the study has been changed to “internalization”.

*2.* *Introduction:*

*Lines 63-68 contradict the presence of H. pylori in gastrointestinal tract, including the oral cavity. According to several reports mentioned in the present study, occurrence of H. pylori has been demonstrated in the oral cavity by detection of H. pylori-specific genes. Furthermore, in the last line of conclusions in the discussion section, authors concluded that H. pylori transmission occurs during vaginal delivery and through the oral cavity of newborn. That means that even Candida yeast (as a shelter for H. pylori) has to enter the gastrointestinal tract of mother or child through the portal of oral cavity. Accordingly, in the present study, detection of H. pylori genes in Candida isolates from oral cavity of mothers and newborns could help to better clarify the results. However, a similar but more elaborated study has been performed and the results have been published in 2013 (reference # 24).*

**Response:** We believe that these contents are not contradictory and are intended to explain the fact that the oral cavity is not a suitable host for the colonization of *Helicobacter pylori*.

*3. Line 68-69 is confusing and needs to be revised and supported by a reference.* *Line 73 is confusing. Line 77-78 must be clarified.* *Lines 76-84 must be included in one paragraph as the aim of study.*

**Response:** *Line 68-69 is confusing and needs to be revised and supported by a reference.* Mao *et al.* presented a critical discussion of previous studies investigating the potential colonization of the oral cavity by *H. pylori* [1].

*Line 73 is confusing.* The original 73 lines are “However, little attention has been paid to transmission routes for *H. pylori* beyond the typical oral-oral routes or fecal-oral routes.” now modified as "However, there has been little focus on investigating alternate transmission routes for *H. pylori* beyond the commonly recognized oral-oral or fecal-oral routes." *Line 77-78 must be clarified. Lines 76-84 must be included in one paragraph as the aim of study.* Lines 77-78 can be explained by lines 79-84, thus lines 76-78 are placed after lines 84 and in a paragraph.

**Response:** The revised text of the above questions is shown in the manuscript.

*4. Methods: It is not clear why the number of samples in different groups is not equal. Some methods like Live/Dead bacterial staining is too long and some like UBT has not been described. Patients’ information like age and type of gastric diseases is missing. Results of Candida identification and their correlation with other results were not considered in results and discussion sections. The relationship between type of Candida species and detection of intracellular H. pylori genes or observing BLBs has not been considered. What was the origin of the reference C. albicans (ATCC 10231)? The transmission electron microscopy section needs more details. This sentence is not clear and needs explanation: “Candida was treated with 32 μg/mL amphotericin B at 37°C shaking at 120 r/min for 24 h [28]”. What was the purpose of using amphoterici B? For detection of urease activity in Candida: Did you detect this activity in all the yeasts positive for H. pylori? There is no description of recruited yeasts. A reference is needed for SDA+40% urea. Why 40% urea was used? What was the pH of the medium?*

*It is not clear why the number of samples in different groups is not equal.*

**Response:** The number of pregnant women and female patients with digestive disorders varied, as patients came from both outpatient and inpatient units and had relatively large mobility.

*Patients’ information like age and type of gastric diseases is missing.*

**Response:** Data are not available for public access due to patient privacy concerns, but datasets generated during the current study are available from the corresponding author upon reasonable request.

*Some methods like Live/Dead bacterial staining is too long and some like UBT has not been described. Patients’ information like age and type of gastric diseases is missing. Results of Candida identification and their correlation with other results were not considered in results and discussion sections. The relationship between type of Candida species and detection of intracellular H. pylori genes or observing BLBs has not been considered. What was the origin of the reference C. albicans (ATCC 10231)? The transmission electron microscopy section needs more details.*

**Response:** An activity assay staining time of 15 min was used to allow adequate staining of *Candida* cells and their intracellular structures. *Candida albicans* standard strain ATCC10231, a strain deposited in our laboratory. The revised text of the above questions is shown in the manuscript.

*5. Results: 1- “Furthermore, among H. pylori-negative patients, only one vaginal Candida isolate lacked BLBs and was negative for H. pylori 16S rDNA and ureA (Fig 1). These results indicate that H. pylori 16S rDNA was present in Candida isolates with BLBs. Individuals who provided such specimens were therefore infected with H. pylori”.*

*To correlate the detection of 16S rDNA or intracellular bacteria inside the vaginal yeast to H. pylori infection in the stomach or positive IgG antibody, strong explanations and supporting references are needed. Results of this study do not support this conclusion.*

**Response:** Although only one *Candida* isolate, the vaginal *Candida* isolate of *H. pylori*-negative patients, lacked BLBs and was negative for *H. pylori* 16S rDNA and urea, so we need to do a lot of research on it in the future. In addition, thirteen vaginal *Candida* isolates from pregnant women, who were positive for *H. pylori* IgG antibody, were positive for intracellular *H. pylori* specific 16S rDNA.

*Results: 2-This must become clear in this study that why the authors subcultured yeast isolates more than 10 times. These subsequent subculturings (on SDA+ chloramphenicol) must have been performed at the beginning of isolation of yeasts and from then on, there is no need to subculture more than 10 times because the primary purpose should have been removing any probable extracellular bacterial contamination.*

**Response:** To eliminate any possible bacterial contamination, yeast isolates were sub-cultured on SDA medium with chloramphenicol ten times starting from the sample inoculation.

*Results: - What was the difference between BLB-positive and BLB-negative Candida yeasts?*

**Response:** BLB-positive *Candida* refers to the presence of *Helicobacter pylori* genes and antigens in the *Candida*, and conversely, BLB-negative *Candida* refers to the absence of *H. pylori*.

*Figure 4: Light microscopy: The images are so small and unclear that one cannot differentiate between bacterial cell and other intracellular entities. Arrows do not really point to any clear entity. These photographs do not show any convincing results.*

*Figure 5: Live/Dead stain: There is no need to describe the mechanism of staining in the legend. Arrows in B could point to yeast’s nucleus or mitochondria which generally stain green. There is no mention of killing yeast in method section, also no comment on the control yeast that stained green.*

*Figure 6: Micrographs show that yeasts’ specimens were not properly prepared. They show no details of intracellular structures. “High electron density body” is confusing and needs to be explained.*

*Fig 7: Photograph C must be replaced by a better one with higher magnification.*

*Figures in this section that are the main part of this study fail to show convincing results.*

**Response:** The figures and contents of Figs. 4–7 have been corrected for the review comments, see the corresponding sections in the manuscript.

*6. Discussion:*

*Line 249-250: Need a reference. Line 252-253: Authors need to specify transmission to newborns’ oral cavity through vaginal delivery. Line 256: The importance of cagA positivity has not been discussed. Line 261-262: “H. pylori- specific nucleic acids” is not consistent with other parts of the manuscript. Line 272-273: Use of amphotericin must be explained. Line 278-279: References Burgers et al. 2008, Siavoshi &Taghikhani 2013 and Salmanian et al. 2008 are not related and have been misused.*

*Line 284-287: Are these related to your study or need a reference. In this study only two gastric yeasts were used that is not convincing and do not correlate with vaginal yeasts. Line 288-292: Not included in the method and results of this manuscript. Furthermore, references are not relevant and have been misused. Line 294-299: These lines are not related to the study and have been used by mistake. Line 300-304: Totally misinterpreted and the references are not related.*

**Response:** References have been added to Line 249-250. The remaining modifications are shown in the manuscript.

*Line 288-292: Not included in the method and results of this manuscript.* Under light microscopy, we observed rapid movement of bacteria within *Candida* vacuoles, and we can provide the video as Supplementary Material.

*Line 294-299: These lines are not related to the study and have been used by mistake. Line 300-304: Totally misinterpreted and the references are not related.* For this part of the content, we stick to our point of view without making changes.

**Response:** The revised text of the above questions is shown in the manuscript.

**Reviewer #4:** *I have only a minor suggestion regarding the discussion. Convincing evidence of intracellular survival of H. pylori has been also published for some Entamoeba species (see, particularly, the work of Ferrus et al.). Probably this should be mentioned in the discussion, as it suggests that the capacity to survive in eukaryotic cell may not be limited to amebas or yeasts.*

*Furthermore, I found very interesting that Candida species carrying H. pylori showed detectable urease activity. Might this fact suggest a symbiotic relationship between H. pylori and Candida allowing the yeast to survive in acidic environments?*

**Response:** *H. pylori* is known to be present in amebas or yeasts, and probably in other eukaryotic cells.

*Candida albicans* is an opportunistic fungal pathogen that can colonize host niches at varying pH [2]. *Candida* species carrying *H. pylori* showed detectable urease activity. It suggests a symbiotic relationship between *H. pylori* and *Candida*, allowing the yeast to survive in acidic environments.

1.	Mao X, Jakubovics NS, Bachle M, Buchalla W, Hiller KA, Maisch T, et al. Colonization of Helicobacter pylori in the oral cavity - an endless controversy? Crit Rev Microbiol. 2021;47(5):612-29. Epub 2021/04/27. doi: 10.1080/1040841X.2021.1907740. PubMed PMID: 33899666.

2.	Vacca I. Fungal physiology: Acidic pH interferes with Candida persistence. Nature reviews Microbiology. 2017;15(7):382. Epub 2017/06/14. doi: 10.1038/nrmicro.2017.72. PubMed PMID: 28607379.
